# Stress, mental health, and resources of palliative care professionals

**DOI:** 10.1017/S1478951524002050

**Published:** 2025-03-28

**Authors:** Isabelle Cuchet, Axelle Maneval, Michael Dambrun

**Affiliations:** 1 Laboratory of Social and COgnitive Psychology (LAPSCO) UMR CNRS 6024, University Clermont Auvergne, Clermont-Ferrand, France; 2 UR ACCePPT, University Clermont Auvergne, Clermont-Ferrand, France; 3 Unity of Palliative Care, CHU Clermont-Ferrand, Clermont-Ferrand, France

**Keywords:** Palliative care professionals, secondary traumatic stress, acute and chronic stress, psychological resources, self-compassion, psychological flexibility

## Abstract

**Objectives:**

Palliative care (PC) professionals confront the suffering and mortality of their patients, adding to the conventional stressors related to work dynamics or personal life. However, the specific stressors inherent in end-of-life (EOL) care and their relation with the mental health of these professionals, remain inadequately explored. This study seeks to examine the respective roles of various stressors encountered by PC professionals and their associations with mental health. Additionally, it aims to elucidate the relationships between specific psychosocial factors (psychological resources or work environment perceptions) and mental health within the context of stress associated with PC.

**Method:**

An online questionnaire was developed and distributed to PC professionals in France (e.g., doctors, nurses, care assistants, psychologists). The questionnaire contained measures of stress experienced in the last 6 months (personal, professional, or EOL); measures of mental health; and measures of psychosocial factors (perceptions of work environment and psychological resources).

**Results:**

Three hundred and seventy-nine participants completed the entire questionnaire in November 2022. Among the various stress factors assessed, the accumulation of EOL care emerged as a robustly linked stressor to poorer mental health. In this specific context of EOL care stress, self-compassion and psychological flexibility are significantly related to well-being and mental health, even when other psychosocial factors related to the work environment are statistically controlled.

**Significance of results:**

This study is the first to highlight the main stressor affecting the well-being of PC professionals – the accumulation of EOL care – along with the key resources – psychological flexibility and self-compassion – that are associated with their well-being.

## Introduction

Maintaining or enhancing the well-being of palliative care (PC) professionals is a priority in a global context where the need for end-of-life (EOL) care is increasing, particularly as populations age. Every day, hospice professionals are exposed to the suffering and death of individuals. Outside of their professional realm, being confronted with the suffering and death of others is considered a potentially traumatic event that can lead to post-traumatic stress disorder (PTSD), characterized by symptoms such as avoidance, intrusive thoughts, flashbacks, anger, or anxiety (American Psychiatric Association 2013). This syndrome has also been reported among PC professionals (O’Mahony et al. 2016). A systematic review of the literature on the prevalence of burnout in this population also showed that 17% of PC professionals suffer from it (Parola et al. 2017), making it a particularly vulnerable profession. Several studies have also established the presence of symptoms of anxiety, depression, or compassion fatigue (CF) in these same professionals (Melvin 2015; O’Mahony et al. 2016; Samson and Shvartzman 2018). CF, defined as a secondary traumatic reaction resulting from close contact with the suffering or trauma of others (Figley 1995; Slocum-Gori et al. 2013), mirrors many symptoms of PTSD. Authors such as Bride et al. (2007) and Melvin (2015) have also noted exhaustion and a diminished ability to care for others as additional symptoms. While post-traumatic stress and burnout can affect any individual, CF is specific to helping contexts and manifests in professionals who are in direct contact with the suffering of others, such as healthcare professionals and police officers. Finally, a meta-analysis concludes that working in PC puts the overall well-being of healthcare professionals at risk (Zanatta et al. 2020). However, amidst these challenges, some authors (e.g., Sansó et al. 2015; Sinclair 2011; Slocum-Gori et al. 2013) have highlighted positive signs of well-being among EOL professionals, including the emergence of compassionate satisfaction. Compassionate satisfaction is defined as the gratification derived from assisting others during times of suffering (Galiana et al. 2020).

The myriad studies assessing the well-being of doctors and nurses repeatedly exposed to dying patients have yielded inconsistent results (Samson and Shvartzman 2018). These factors underscore the need for a closer examination of the various stressors faced by EOL professionals and their effects on mental health. Some studies have begun to address this issue, yet the term “stressor” or “stress factor” typically encompasses a broad range of variables, making comparisons difficult. Reports indicate that stressors for PC professionals include the work environment, role conflict, repeated exposure to death, inadequate time spent with dying patients, heavy workloads, inadequate coping mechanisms for their own emotional responses to death, communication difficulties with patients or families, and feelings of anger, depression, or guilt (Kearney et al. 2009; Peters et al. 2012).

The term “stressor” encompasses any experience in which the environmental demands of a situation exceed the psychological and/or physiological capacity to cope effectively (Cohen et al. 2016). However, accurately measuring this broad concept remains challenging. A recent guide to good practice in stress measurement has been published (Crosswell and Lockwood 2020). Authors advocate for distinguishing between exposure to potentially stressful events and individuals’ responses to these exposures. Since the same event does not necessarily impact all individuals similarly, it is crucial to assess perceived stress levels beyond the number of potentially stressful events experienced. The duration of the stressful event is also crucial to evaluate, as acute stress (exposure to a threatening situation over a short period) may not have the same impact on health as chronic stress (exposure to threatening circumstances over a prolonged period of at least 1 month) (Crosswell and Lockwood 2020). For PC professionals, exposure to the suffering and death of others can be considered as acute stress if professionals feel challenged by 1 or several specific situations, or as chronic stress if they struggle to cope with the exposure to EOL scenarios over an extended period. As of now, no study has thoroughly investigated these various factors.

The mental well-being of individuals facing stressful situations is intertwined with various psychosocial factors, both environmental and personal. Within an occupational context, Karasek’s job demand-control-support JDCS model (Karasek et al. 1998) stands as one of the most robust theoretical frameworks for linking certain psychosocial factors to mental health. This model posits that increased job demands heighten professionals’ stress levels, while job decision latitude (the sense of being able to develop one’s skills or make decisions, for example) and social support (such as relationships with colleagues and the perception that others make work easier) act as protective factors for well-being. In the specific context of PC, job demands, social support, and feelings of competence have been linked to job satisfaction and/or distress (e.g., Fillion et al. 2007; McCloskey and Taggart 2010; Papworth et al. 2023). On a personal level, while coping strategies have long been central to stress management research (Folkman and Lazarus 1988), more recent attention has shifted toward psychological resources mobilized by individuals. One such dispositional resource is mindfulness, or the attention to the present moment. Mindfulness has been defined as the awareness that arises from intentionally paying attention, in the present moment and without judgment, to the flow of experience (Kabat-Zinn 2003). This disposition, which promotes the subjective observation of thoughts, emotions, and physical sensations, can be cultivated through various meditation and psychoeducational techniques (Kabat-Zinn 2009). The propensity for mindfulness has been recognized as a source of well-being and adaptation among healthcare professionals (Conversano et al. 2020). Some studies have shown that mindfulness-enhancing interventions are particularly effective in reducing burnout and improving compassionate satisfaction among PC professionals (e.g., Orellana-Rios et al. 2018). Furthermore, some authors argue that mindfulness-based interventions can enhance focus on others and self-compassion among healthcare professionals (Boellinghaus et al. 2014; Sansó et al. 2015). Self-compassion can be defined as the capacity to acknowledge one’s own suffering without avoidance or detachment, coupled with the intention to alleviate it. It involves extending nonjudgmental understanding toward one’s own pain, inadequacies, and failures, recognizing them as part of the broader human experience (Neff 2003). This definition encompasses 3 key components: benevolence (replacing self-judgment), mindfulness (instead of identifying with negative thoughts or emotions), and common humanity (contrasting with feelings of isolation due to personal problems or shortcomings). Recent studies have highlighted the practice of self-compassion among PC professionals as a means to better cope with the suffering and death of others, thereby enhancing their overall quality of life (Galiana et al. 2020; Orellana-Rios et al. 2018). Moreover, self-compassion and mindfulness are closely associated with psychological flexibility, which broadly refers to an individual’s capacity to act in alignment with their values while acknowledging and accepting the occurrence of challenging psychological events (Kashdan and Rottenberg 2010). Conversely, psychological inflexibility describes difficulties in aligning behavior with values and goals due to struggles in engaging with the environment and managing distressing thoughts or emotions. Generally, experiential avoidance and psychological inflexibility exhibit negative correlations with well-being (Hayes et al. 1999). For instance, a recent study on geriatric nurses in Spain found that psychological flexibility serves as a buffer against stress, fostering compassionate satisfaction and reducing levels of CF (Sarabia‐Cobo et al. 2021). Additionally, empathy plays a central role in the patient-professional relationship within PC. Empathy arises when observing or imagining another individual’s emotional state triggers a similar affective response (Duarte and Pinto-Gouveia 2017). While empathy is crucial for establishing therapeutic relationships in healthcare settings, excessive sensitivity to patient suffering can lead to detrimental outcomes, including CF (Bride et al. 2007). Particularly in PC, empathy may incur significant costs in terms of professional well-being (Cross 2019).

The primary objectives of this study were 2-fold: first, to examine the respective roles of various stressors encountered by PC professionals and their associations with mental health; second, to explore the relationships between psychological resources utilized in the context of stress related to PC management and well-being, while controlling for other psychosocial factors (e.g., job demand, social support, job decision latitude, as outlined in Karasek et al. 1998).

## Method

### Data and procedure

Participants were recruited primarily by email through the Société Française d’Accompagnement et de soins Palliatifs online directory, which includes contacts for all PC facilities (around 10,000 professionals) throughout France. Questions were administered online using Qualtrics survey software. The survey remained open to respondents between October 19, 2022, and December 1, 2022. This work was approved by the IRB-UCA Research Ethics Committee, under number IRB00011540-2022-30, in accordance with French policy on individual data protection. Participants’ consent was obtained prior to administering the questionnaire.

Participants were PC professionals working in France, whether in hospitals, medico-social establishments or at home. A total of 603 PC professionals clicked on the link to the survey. The survey was designed in such a way that if a question was not answered, the participant would not be able to continue the study (except for free comments). Missing data are therefore not arranged randomly, and the data imputation method is not appropriate in this case (Allison 2009). Of the 603 potential study participants, 379 completed the entire questionnaire. We used these 379 responses for the analyses presented here.

### Measures

The questionnaire contained 33 questions or scales in French, and measured participants’ subjective perceptions of: the stress they had experienced; certain psychosocial factors (linked to their work environment and their own psychological resources); and their mental health. Sociodemographic information (e.g., age, gender, and years of experience) was collected at the end of the questionnaire.

#### Potential stressors perceptions

Psychological perceptions of 8 potential stress factors were measured: perception of stress related to working conditions (i.e. “over the past in the last 6 months, working conditions in my workplace have been very stressful”), perceived stress related to relationships with colleagues, perceived stress related to managing the health crisis at personal and professional levels, perceived personal stress, and perceived stress related to EOL care in the last 6 months, whether this stress was related to a particular care (i. e. “in the last 6 months, 1 end-of-life care has been very stressful”), several care situations (i.e. “in the last 6 months, several end-of-life care situation have been particularly stressful for me”), or the accumulation of care situations (i.e. “in the last 6 months, the accumulation of EOL care situations has been very stressful for me”). Participants were asked to respond on a Likert scale ranging from 1 (strongly disagree) to 7 (strongly agree).

#### Psychosocial factors

Five items from Karasek’s questionnaire (Karasek et al. 1998) were selected, relating to 3 dimensions: job demand, job decision latitude (divided into 2 subdimensions: the feeling of being able to develop personal skills at work and the decision-making latitude enjoyed), and perceived social support, through relationships with colleagues and the feeling that people around you are task facilitators. An item on identification with one’s work was also included, as well as a measure of the amount of work time devoted to EOL or PC in the last 6 months.

Four psychological resource scales were also selected.

*Empathy*. The short Basic Empathy Scale: French version, (Jolliffe and Farrington 2006), shortened to 12 items, was used. This scale focuses on 3 dimensions of empathy: emotional contagion, cognitive empathy, and emotional disconnection. Individuals are asked to indicate their degree of agreement with each of the propositions, from 0, “strongly disagree,” to 4, “strongly agree.” The scale, validated in French, comprises 20 items. We selected the 12 items with the highest factor loadings (Carré et al. 2013) (ω McDonald = .75, see Supplementary Materials, Table S1).

*Attention to the present moment.* The 15-items Five-Facets Mindfulness Questionnaire was used; this questionnaire, validated in French in its long version (39 items), is one of the most common for measuring mindfulness or attention to the present moment. The scale assesses 5 dimensions of resourcefulness: the ability to observe sensations, perceptions, thoughts and feelings; the ability to describe lived experience; the ability to act mindfully; the absence of immediate reaction to inner experience; and the absence of judgment of inner experience. The person is asked to answer the items on a scale from 1 (“never or very rarely true”) to 5 (“very often or always true”). We first used the 15 French items of the short version validated in English (Baer et al. 2008). In our study, item 6 “I notice how food and drink influence my thoughts, body sensations and emotions” did not correlate with the rest of the scale (item-rest correlation = 0.02). Thus, this item has been removed. A new 14-item subscale was used for further analysis, without this item (McDonald’s ω = .80, see Supplementary Materials, Table S2).

*Self-compassion.* The Short Self Compassion Scale was used in a shortened 7-item version. The 7 items with the highest factor loadings from the short version validated in English (Raes et al. 2011) were selected. This questionnaire, validated in French in its original version, assesses 5 dimensions of self-compassion: self-kindness (1 item), nonjudgment of self (2 items), isolation (2 items), mindfulness (1 item), and overidentification (2 items). The participant is asked to answer how often he or she behaves in the way suggested, from 1 (“almost never”) to 5 (“almost always”). (McDonald’s ω = .85, see Supplementary Materials, Table S3).

*Psychological flexibility.* The French version of the Multidimensional Psychological Flexibility Inventory, (Grégoire et al. 2020), in a shortened 12-item version, was used. In its complete version, this scale assesses an individual’s capacity for acceptance and commitment. Twelve dimensions of flexibility (6 items) and inflexibility (6 items) are assessed: acceptance, contact with the present moment, observer-self abilities, defusion, recognition of one’s values, committed action, experiential avoidance, loss of contact with the present moment, self as content, fusion, loss of contact with one’s values, and inaction. The participant is asked to answer how often he or she behaves in the way suggested, from 1 (“almost never”) to 7 (“almost always”). We selected 12 items from version 24, validated in French by Grégoire, retaining only 1 item per dimension instead of 2 in Grégoire’s version, in order to shorten the time required for participants to complete the questionnaire. However, after an initial internal consistency analysis, item 7 related to experiential avoidance (“I tried to distract myself when I felt unpleasant emotions”) showed negative correlations with the rest of the inflexibility component of the scale (item-rest correlation = −.33) and was removed. A new 11-item subscale, without this item, was used for further analysis. The 11-items scale retains acceptable internal consistency compared with the 12-items scale (McDonald’s ω = 0.84, see Supplementary Materials, Table S4).

#### Mental health

Single-item visual analog scales (VASs) have been successfully used to assess a wide variety of health-related concepts, including quality of life and mood (de Boer et al. 2004; Williams et al. 2010). One advantage of VAS is that they are brief, simple to administer and not very demanding for the respondent. These characteristics make them an ideal tool for lengthy questionnaires such as ours. We used 5 VASs to measure feelings of well-being such as feelings of inner peace, anxiety, life satisfaction, depression, and happiness (Bonacchi et al. 2021; Zeidan 2012). These scales allow participants to indicate their level of well-being along a continuum from 1 to 7 (McDonald’s ω = 0.84, see Supplementary Materials, Table S5). Finally, we measured 3 other dimensions of well-being via the Short Professional Quality of Life scale (Galiana et al. 2020; Stamm 2010). This 9-item scale assesses 3 dimensions of quality of life for professionals working with individuals in distress: compassion satisfaction, defined as “the pleasure of being able to do one’s job well” (helping others), burnout defined as “feelings of hopelessness and difficulties in coping with work or performing it effectively,” and CF, considered in this scale as a synonym of secondary traumatic stress (STS) and defined as “a set of difficulties linked to secondary exposure to people who have experienced extremely stressful events” (Bride et al. 2007). Participants are asked to determine the frequency of occurrence of different symptoms over the past 30 days from 0 to 12 (McDonald’s ω = 0.82, see Supplementary Materials, Table S6).

### Statistical analysis

Data were processed using an Excel spreadsheet and Jamovi and JASP statistical software. Statistical significance was considered at 0.05 for all analyses. A factorial analysis of the various mental health measures collected was first carried out to verify the number of factors actually measured using the 8 scales used, and to reduce the number of variables if necessary by grouping some of them together. The links between potential stressors and mental health were explored using correlations, then multiple regressions to test the relevance of each link between stressors and mental health. To explore the links between the various psychosocial factors measured and mental health in a context of EOL care stress, we selected the EOL care stressor most closely related to the various mental health measures. We performed simple linear regression analyses to determine expected levels of mental health as a function of stress levels among all participants. For each participant, the residual between their expected mental health score (the result of the regression) and their actual score can be interpreted as an under or overreaction to the stressor relative to all participants (see Amstadter et al. 2014; Veer et al. 2021). The links between these under or overreactions and various environmental and personal factors (job demand, job decision latitude, perceived social support, self-compassion, empathy, psychological flexibility, attention to the present moment) were explored using correlation and then multiple regression analyses.

## Results

### Sociodemographic profile of participants

Participants ranged in age from 23 to 69, with an average age of 45.7 (10.4) years. The majority of participants are women (86%). Participants have an average of 10.1 (7.37) years’ experience in the position, and spend 28.7 (11.2) hours per week in EOL or PC. Participants are mainly nurses (33.8%), physicians (30.3%), psychologists (18.2%) and nursing aides (5.8%) working in PC units or mobile teams. The remaining 11.9% held various roles, including occupational therapists, health executives, physiotherapists, psychometricians, art therapists, dieticians, socio-estheticians, and chaplains.

### Mental health measure

Table 1 presents the mental health scores of participants. We had no hypothesis on the number and nature of latent factors present within our mental health measures. Thus, following the protocol recommended by Taherdoost et al. (2022), an exploratory factor analysis was first carried out to identify the number of dimensions of our overall measures, and to reduce the number of variables if necessary. As our data were not normally distributed, we chose the principal axis factoring method to extract the factors. The Kaiser-Meyer-Olkin measure of sampling adequacy is .81, and Barlett’s test of sphericity is significant (*p* < .001). As recommended (Taherdoost et al. 2022), we performed a parallel analysis to select the number of factors to be retained. The chi-squared was significant (*p* < .001). The Tucker Lewis index was equal to .94, indicating a good adjustment (Byrne 1994). The root mean square error of approximation was estimated to be 0.0810 (90% CI [.046, .124]), indicating that the model can be considered adequate (Byrne 1994). The results presented in Table 2 indicate that the set of measures can be reduced to 3 factors.

**Table 1.** Mental health measures of participants

		95% confidence interval Mean						
	Mean	Upper	Lower	Min.	Max.	25th percentile	50th percentile	75th percentile
Life satisfaction	5.33	5.44	5.21	1.00	7.00	5.00	5.50	6.00
Anxiety	3.50	3.67	3.33	1.00	7.00	2.00	3.00	5.00
Happiness	5.26	5.38	5.13	1.00	7.00	5.00	5.00	6.00
Depression	2.40	2.56	2.24	1.00	7.00	1.00	2.00	4.00
Inner peace	4.53	4.69	4.37	1.00	7.00	3.50	5.00	6.00
Burnout	3.87	4.15	3.60	0.00	12.00	2.00	4.00	6.00
Compassion satisfaction	10.74	10.91	10.57	0.00	12.00	10.00	11.00	12.00
Compassion fatigue	3.12	3.34	2.90	0.00	12.00	1.00	3.00	4.00

**Table 2.** Results from a factor analysis of the 8 mental health measures

Mental health measures	Factor loading		
	1	2	3
Life satisfaction	.80		
Anxiety		.66	
Happiness	.87		
Depression		.67	
Inner peace	.39		
Burnout			.82
Compassionate satisfaction	.43		
Compassion fatigue			.67

***Note***. The “Principal axis factoring” extraction method was used in conjunction with a “obliminvarimax” rotation.

For further analysis, we created 3 new mental health variables, the “well-being” variable corresponding to the average of the scores for the “life satisfaction,” “happiness,” “inner peace” and “compassionate satisfaction” variables, the “burnout-CF” variable corresponding to the average of the burnout and CF scores, and the “anxiety-depression” variable corresponding to the average of the anxiety and depression scores.

### Relation between mental health, sociodemographic factors, and stressors

As stress perception measures were not normally distributed, we conducted Spearman correlation analyses between mental health measures and stress perceptions in the 6 months preceding the survey. The results are summarized in Table 3.

**Table 3.** Spearman rho correlation coefficients between mental health components and potential stressors experienced in the last 6 months

Potential stressors	Well-being	Burnout-CF	Anxiety-depression
Work conditions	−.21***	.40***	.36***
Colleague relations	−.26***	.30***	.31***
COVID-19 in the professional context	−.14**	.29***	.31***
Personal stress	−.27***	.22***	.38***
COVID-19 in the personal context	−.21***	.28***	.32***
1 EOL care management	−.21***	.42***	.30***
Several EOL care management	−.24***	.45***	.31***
Accumulation of EOL care management	−.32***	.56***	.38***

***Notes***.

** *p* < .01

*** *p* < .001

EOL = end-of-life.

As all potential stressors were significantly correlated with the 3 mental health components, we performed multiple regression analyses to clarify the links between each of these stressors and mental health, when controlling for the others. Among the prerequisites for carrying out these analyses, the hypothesis of noncollinearity between the predictors was verified using the variance inflation factor (VIF). The hypothesis of noncollinearity is considered valid if all VIFs are less than 5 (O’brien 2007). For the Burnout-CF (Burnout-compassion fatigue), anxiety-depression and well-being, all VIFs are below 5. As the normality of the distribution of the different variables was not respected, bootstrap regressions were carried out (here bootstrapping based on 5000 draws) (see Wright et al. 2011). The results are summarized in Table 4.

**Table 4.** Unstandardized bootstrap coefficient and confidence interval (95%) between various potential stressors experienced in the last 6 months (iv) and mental health components (dv)

	Unstandardized β (confidence interval 95%)		
Potential stressors	Well-being	Burnout-CF	Anxiety-depression
Work conditions	−.06 [−.14, .01]	.15 [.07, .24]	.16 [.07, .27]
Colleague relations	−.07 [−.14, − .01]	.03 [−.08, .10]	.08 [−.01, .17]
COVID − 19 in the professional context	.05 [−.03, .12]	.02 [−.06, .1]	.02 [−.08, .12]
Personal stress	−.08 [−.13, − .03]	−.001 [−.05, .05]	.15 [.08, .22]
COVID − 19 in the personal context	−.04 [−.11, .04]	−.01 [−.08, .08]	.05 [−.05, .15]
1 EOL care management	.01 [−.07, .08]	.01 [−.07, .1]	.001 [−.11, .13]
Several EOL care management	.05 [−.04, .14]	−.02 [−.12, .08]	−.08 [−.23, .05]
Accumulation of EOL care management	−.12 [−.21, − .04]	.29 [.20, .38]	.19 [.07, .32]

***Notes***. EOL = end-of-life; bootstrap coefficient estimated on the median of the bootstrap distribution.

Among the 8 proposed sources of stress experienced by participants in the 6 months prior to the study, whether personal, professional or related to EOL or PC management, stress due to relationships with colleagues remains negatively related to well-being (β = −.07; 95% CI: −.14,−.01). Stress due to working conditions remained positively related to Burnout-CF (Burnout-compassion fatigue) (β = .15; 95% CI: .07, .24) and anxiety-depression (β = .16; 95% CI: .07, .27). Personal stress experienced in the last 6 months remained negatively related to well-being (β = −.08; 95% CI: −.13, −.03), and positively to anxiety-depression (β = .15; 95% CI: .08, .22). Stress due to the accumulation of EOL care management remained positively related to Burnout-CF (Burnout-compassion fatigue) (β = .29; 95% CI: .20, .38), to anxiety-depression (β = .19; 95% CI: .07, .32), and negatively to well-being (β = −.12; 95% CI: −.21, −.04). It is the only factor that remains significantly related to all 3 mental health measures, and specifically targets a type of stress related to EOL care. The other EOL stressors (i.e., stress related to 1 specific EOL care or stress related to several specific EOL care) are not significantly related to mental health measures when the other stressors are statistically controlled.

### Relations between psychosocial factors and mental health in a context of stress linked to EOL care

The following analyses focus on the links between psychosocial factors and mental health in a context of EOL care stress. Our main stressor of interest (i.e. cumulative EOL care management) emerges as one of the stressors robustly linked to mental health (see Table 4). We therefore selected this stressor for further analysis. Following the procedure outlined by Amstadter et al. (2014), we first performed 3 simple linear regressions to identify the link between this stressor and each of the 3 mental health components. These regression functions indicate expected mental health scores as a function of stress level, across all participants. For each participant, the residual between their regression score and their actual mental health score can be interpreted as their over or underreaction to stress on that mental health component, relative to other participants. If the component is of negative valence (burnout-CF and anxiety-depression), a negative residual indicates the participant’s underreaction to the stressor, as the regression function linking the stressor to the component is increasing (β > 0). If, on the other hand, the mental health component is of positive valence (well-being), the underreaction appears with a positive residual, as the regression linking the stressor to the well-being measure is decreasing (β < 0). The links between these residuals and the various psychosocial factors measured (job demand, feeling of being able to develop one’s skills at work, decision latitude, feeling that people make the task easier at work, quality of relationship with colleagues, identification with one’s work, self-compassion, empathy, psychological flexibility, attention to the present moment) were in turn explored by means of correlation analyses and then multiple regression. As the residuals were not normally distributed, we used Spearman’s rho for the correlation analyses presented in Table 5.

**Table 5.** Spearman rho correlation coefficients between psychosocial factors and residuals (participants’ over or underreactions to the stressor) on the 3 mental health measures

Psychosocial factors	Residual well-being regression	Residual burnout-CF regression	Residual anxiety-depression regression
Feeling of being able to develop one’s skills at work	.28***	−.22***	−.18***
Decision latitude	.19***	−.22***	−.14**
Feeling that people make the task easier at work	.32***	−.20***	−.20***
Identification with one’s work	.04	.16***	.04
Quality of relationship with colleagues	.21***	−.14***	−.14**
Job demand	.01	.07	.07
Attention to the present moment	.35***	−.34***	−.45***
Self-compassion	.36***	−.41***	−.50***
Psychological flexibility	.47***	−.39***	−.48***
Empathy	.007	.11*	−.12*

***Notes***.

* *p* < .05

** *p* < .01

*** *p* < .001

EOL = end-of-life.

With the exception of the feeling of having a heavy job demand, all the psychosocial factors measured are significantly correlated with the residuals of the simple regression between the burnout-CF and the stress of accumulating EOL care. These psychosocial factors are positively correlated with participants’ underreaction to this stressor (negative correlations with the residuals), except for the sense of identification with one’s work and empathy, which are positively correlated with participants’ overreaction to this stressor. On the Well-being component, with the exception of the feeling of having a heavy job demand, the feeling of identification with one’s work and empathy, the psychosocial factors measured are significantly correlated with the residuals of the simple regression between well-being and the stress of accumulating EOL care. These psychosocial factors are positively correlated with participants’ underreaction to this stressor. Finally, on the anxiety-depression component, with the exception of the feeling of having a heavy job demand and the feeling of identification with one’s work, the psychosocial factors measured are significantly correlated with the residuals of the simple regression between anxiety-depression and the stress of accumulating EOL care. These psychosocial factors are positively correlated with participants’ underreaction to this stressor (negative correlations with residuals).

We performed multiple regression analyses to identify psychosocial factors robustly related to participants’ over or underreactions to the stressor, statistically controlling for other variables. All VIFs were below 3. As the normality of the distribution of the measures was not respected, we conducted bootstrap regressions (bootstrapping based on 5000 draws). The results are summarized in Table 6.

**Table 6.** Unstandardized bootstrap coefficient and confidence interval (95%) between psychosocial factors (iv) and mental health components (dv)

	Unstandardized β (confidence interval 95%)		
Psychosocial factors	Residual well-being regression	Residual burnout-CF regression	Residual anxiety-depression regression
Feeling of being able to develop one’s skills at work	.06 [.002, .13]	−.01 [−.08, .05]	.01 [−.06, .09]
Decision latitude	−.04 [−.1, .02]	−.04 [−.1, .03]	.04 [−.04, .11]
Feeling that people make the task easier at work	.09 [.03, .16]	−.09 [−.13, .04]	−.08 [−.19, .01]
Identification with one’s work	-	.09 [.04, .13]	-
Quality of relationship with colleagues	−.001 [−.08, .09]	−.03 [−.15, .07]	.01 [−.11, .12]
Job demand	-	-	-
Attention to the present moment	.001 [−.02, .01]	−.01 [−.03, .004]	−.03 [−.05, − .006]
Self-compassion	.02 [−.000, .03]	−.03 [−.05, − .01]	−.06 [−.09, − .04]
Psychological flexibility	.04 [.03, .06]	−.02 [−.04, − .01]	−.03 [−.06, − .01]
Empathy	-	<.001 [−.02, .02]	.01 [−.01, .03]

***Notes***. EOL = end-of-life; bootstrap coefficient estimated on the median of the bootstrap distribution.

Among all the psychosocial factors measured, self-compassion is significantly linked to an underreaction to stress on both burnout-CF (β = −.03; 95% CI: −.05, −.01)) and anxiety-depression (β = −.06; 95% CI: −.09, −.04) factors. Flexibility is significantly related to an underreaction to stress on the 3 factors burnout-CF (β = −.02; 95% CI: −.04, −.01), well-being (β = .04; 95% CI: .03, .06) and anxiety-depression (β = −.03; 95% CI: −.06, −.01). Attention to the present moment was significantly related to stress underreaction only on the anxiety-depression factor (β = −.03; 95% CI: −.05, −.006). Empathy was neither significantly nor trendily related to under or overreaction to stress, whatever the mental health factor studied. On the other psychosocial factors measured, the feeling of identification with one’s work is significantly linked to an overreaction to stress on the burnout-CF component (β = .09; 95% CI: .04, .13). The feeling of being able to develop one’s skills at work (β = .06; 95% CI: .002, .13) and the feeling that people make the task easier at work (β = .09; 95% CI: .03, .16) are both linked to an underreaction to stress on the well-being component.

## Discussion

Our survey aimed to assess the mental health of PC professionals through a 1-time questionnaire, focusing on their experiences of stress and various psychosocial factors. The results provide insights into several factors associated with the well-being of PC professionals. Logically, all stressors encountered in the 6 months preceding the study showed correlations with different mental health metrics (refer to Table 3). Among the 8 stressors presented to participants, the stress related to the accumulation of EOL care in the preceding 6 months emerged as the most closely linked factor to various mental health aspects after accounting for other stressors. This stressor consistently correlated with different mental health measures and emerged as the strongest predictor when controlling for other stressors, such as personal life stress, work-related stress, stress from relationships with colleagues, and stress induced by the pandemic context (the survey was conducted toward the end of the COVID-19 pandemic, in November 2022). Additionally, it was found to be the most predictive factor compared to other stressors associated with EOL care, such as stress from individual EOL care situations or stress from multiple isolated cases. These initial findings offer valuable insights into understanding the mental health of PC professionals. It is conceivable that they possess effective coping resources for managing acute stressors, such as dealing with patient suffering and death, but prolonged exposure to these stressors may eventually impact their mental well-being by depleting these resources over time. In a recent model proposed by Bakker and de Vries (2021) regarding burnout and its regulation, the chronicity of work-related stress is highlighted as a central aspect of the distress process. Prolonged exposure to stressors may lead individuals to engage in self-deprecation and inflexible cognitions and behaviors, limiting the range of coping strategies available and facilitating the onset of burnout.

The term of “death competence” has been proposed to describe specialized skill in tolerating and managing patients problems related to dying, death, and bereavement (Gamino and Ritter 2012; Ho Chan and Tin 2012). This competence highlights the importance of the ability to manage not only the suffering and the death of others but also the impact of death on their self, such as their own death-related feelings (Chan et al. 2016). Authors of this model describe how professionals working in fields related to death and dying can cultivate a particular set of skills, referred to as self-competence in death work, and involving the development of personal resources such as optimism, calmness, or genuineness and the ability to manage both the emotional and existential challenges that arise from their work. Echoing this theory, our results underscore the significance of dispositional resources, particularly psychological flexibility, mindfulness, and self-compassion, as significantly correlated to the mental health of PC workers, irrespective of their perceptions of the professional environment (e.g., opportunities for skill development at work, relationships with colleagues, job demands). Psychological flexibility, in particular, was found to be significantly associated with reduced reactions to stress related to accumulating EOL care across various measures of burnout, CF, anxiety, and depression, regardless of perceived social support, autonomy, or competence. Psychological flexibility involves adapting to the changing demands of the environment, including shifting perspectives and balancing desires, needs, or obligations, especially when they conflict (Kashdan and Rottenberg 2010). This concept, recognized as a key component of stress resilience (Bonanno and Burton 2013), forms the basis of acceptance and commitment therapy (ACT) (Hayes et al. 2006), a 3rd-wave cognitive-behavioral therapy. In ACT, psychological flexibility is viewed as the ability to stay present in the moment, observe and accept thoughts, feelings, and sensations, while engaging in actions consistent with one’s values. This resource has been recognized as a transdiagnostic tool that guards against the onset and persistence of a range of emotional disorders, including depression and anxiety (Bryan et al. 2015), PTSD (Meyer et al. 2019), as well as chronic pain (Gentili et al. 2019), while also promoting overall well-being (Duarte and Pinto-Gouveia 2017; Sarabia‐Cobo et al. 2021). It is crucial for PC professionals to find ways to cope with the emotions brought about by their work, such as managing their own grief after the death of patients (Keene et al. 2010) and developing their emotional intelligence to deal with the suffering of patients and bereaved families (Bailey et al. 2011). Developing the practice of acceptance and commitment in this context of supporting EOL patients could help professionals better manage feelings of powerlessness, sadness, or stress related to the accumulation of EOL care responsibilities. Self-compassion and psychological flexibility can be nurtured through various training programs, such as ACT for enhancing flexibility (Hayes et al. 2006) or compassion-based therapy for cultivating self-compassion (Conversano et al. 2020). These programs offer a diverse array of techniques, ranging from behavioral and cognitive exercises to environmental cues in professional settings, complemented by personalized training sessions (Orellana-Rios et al. 2018).

It is noteworthy that several factors related to the perception of the work environment remain significantly associated with variations in responses to stress from accumulating EOL care even after controlling for dispositional resources. Specifically, identification with one’s work was found to be significantly correlated with professionals’ reactions to burnout or CF (compassion fatigue) symptoms. Social identification, as described by Turner et al. (1987), refers to an individual’s sense of belonging to one or more social groups. While previous research has highlighted the protective effect of work identification against burnout (Avanzi et al. 2018; Correia and Almeida 2020), our findings reveal a negative association between work identification and Burnout-CF in the context of stress related to EOL care. In a meta-analysis of literature concerning group identification and depression, Postmes et al. (2019) have previously found that group identification does not consistently act as a protective factor for well-being among stigmatized groups. Caricati et al. (2022) further observed that a strong identification with one’s work among stigmatized healthcare professionals during the COVID-19 pandemic had a protective effect against burnout but had deleterious consequences on PTSD. This suggests that a negative perception of the professional group could undermine stress management, particularly when individuals strongly identify with the group. Although there is limited research on the stigmatization of PC professionals, the stigma associated with PC itself (Boldt et al. 2006; Zimmermann et al. 2016) might extend to the professionals providing such care. In this situation, professional identification might exacerbate stress instead of acting as a protective factor.

Additionally, perceptions of skill development opportunities at work and facilitation of job tasks by colleagues were both found to be linked to reduced reactions to stress from accumulating EOL care across various measures of well-being. These factors are derived from Karasek’s JDCS model (Karasek et al. 1998), which emphasizes the interplay between psychological job demands, job decision latitude, and social support. The sense of skill development is ingrained within the job decision latitude dimension, while the perception of others facilitating the job aligns with the social support dimension. Across all professions, the opportunity to acquire new skills, foster creativity, and engage in decision-making processes can bolster one’s sense of mastery over their work. PC, often characterized as “all that’s left to do when there’s nothing left to do” by its founder Cicely Saunders (in Aumonier et al. 2017), places particular emphasis on skill development as a cornerstone of professionals’ needs and well-being factors. Additionally, PC is renowned for its teamwork ethos rooted in interdisciplinary collaboration (Frache et al. 2020). Among PC professionals, a culture centered on people, support from colleagues, effective communication, and self-efficacy have been identified as predictors of job satisfaction or emotional distress (e.g., Diehl et al. 2020; McKenna et al. 2022). Our findings regarding the significance of social support for professionals’ well-being strongly resonate with the specific demands of this profession.

Some limitations of our method should be noted. Our first limitation is linked to our statistical analysis based on complete cases only. This type of analysis, which does not add missing data, carries a risk of bias, as the data analyzed may not reflect the general population but only a proportion of professionals, particularly those most affected by work-related stress. Another limitation concerns the timing of the study. The COVID-19 crisis was still ongoing during data collection (November 2022), although the peak of the wave had already passed. This context may have influenced the various measures of perceived stress and mental health among healthcare professionals. Therefore, our findings need to be replicated in a more typical context to ensure greater reliability. A more general limitation is that our sample consisted mainly of female participants. This reflects the reality of the profession in France. It would be important to see if the results could be replicated with male participants and in other cultures, as PC may be dependent on cultural context, particularly for the management of existential suffering. It’s also crucial to acknowledge the cross-sectional nature of this study, which prevents the establishment of causal relationships between the measured variables. Further longitudinal studies that include multiple time assessments of stress, mental health, and resource use among PC professionals are essential for a deeper understanding of the links between death management stressors, mental health and protective resources.

In conclusion, this study underscores the critical need to address the impact of death and dying on the mental health of PC professionals. Constant exposure to suffering and death presents a considerable source of stress, making it essential to provide appropriate support to safeguard their well-being. The findings also emphasize that individual coping resources, such as psychological flexibility can be developed to help manage the chronic stress that arises from confronting suffering and death. Implementing programs aimed at cultivating this skill within PC teams is likely to enhance the well-being of both the professionals and the patients they serve.

## Supporting information

Cuchet et al. supplementary material Cuchet et al. supplementary material

## Supplementary material

The supplementary material for this article can be found at https://doi.org/10.1017/S1478951524002050.

## References

[ref1] Allison PD (2009) *Fixed Effects Regression Models*. Thousand Oaks, USA: SAGE Publications.

[ref2] American Psychiatric Association (2013) *Diagnostic and Statistical Manual of Mental Disorders*, 5th. Washington, DC: American Psychiatric Publishing.

[ref3] Amstadter AB, Myers JM and Kendler KS (2014) Psychiatric resilience: Longitudinal twin study. *The British Journal of Psychiatry* 205(4), 275–280. 24723629 10.1192/bjp.bp.113.130906 PMC4180845

[ref4] Aumonier N, Beignier B and Letellier P (2017) Chapitre III - Les soins palliatifs. *L’euthanasie*. Paris: Presses Universitaires de France, 25–29.

[ref5] Avanzi L, Fraccaroli F, Castelli L, et al. (2018) How to mobilize social support against workload and burnout: The role of organizational identification. *Teaching and Teacher Education* 69, 154–167.

[ref6] Baer RA, Smith GT, Lykins E, et al. (2008) FFMQ-15: 15-item five-facet mindfulness questionnaire. *Assessment* 15, 329–342. 18310597 10.1177/1073191107313003

[ref7] Bailey CJ, Murphy R and Porock D (2011) Dying cases in emergency places: Caring for the dying in emergency departments. *Social Science & Medicine* 73(9), 1371–1377. 21940086 10.1016/j.socscimed.2011.07.036

[ref8] Bakker AB and de Vries JD (2021) Job demands–resources theory and self-regulation: New explanations and remedies for job burnout. *Anxiety, Stress, & Coping* 34(1), 1–21. 32856957 10.1080/10615806.2020.1797695

[ref9] Boellinghaus I, Jones FW and Hutton J (2014) The role of mindfulness and loving-kindness meditation in cultivating self-compassion and other-focused concern in health care professionals. *Mindfulness* 5, 129–138.

[ref10] Boldt AM, Yusuf F and Himelstein BP (2006) Perceptions of the term palliative care. *Journal of Palliative Medicine* 9(5), 1128–1136. 17040151 10.1089/jpm.2006.9.1128

[ref11] Bonacchi A, Chiesi F, Lau C, et al. (2021) Rapid and sound assessment of well-being within a multi-dimensional approach: The well-being numerical rating scales (WB-NRSs). *PloS One* 16(6), e0252709. 10.1371/journal.pone.0252709 PMC8202918 34125831

[ref12] Bonanno GA and Burton CL (2013) Regulatory flexibility: An individual differences perspective on coping and emotion regulation. *Perspectives on Psychological Science* 8(6), 591–612. 26173226 10.1177/1745691613504116

[ref13] Bride BE, Radey M and Figley CR (2007) Measuring compassion fatigue. *Clinical Social Work Journal* 35, 155–163.

[ref14] Bryan CJ, Ray-Sannerud B and Heron EA (2015) Psychological flexibility as a dimension of resilience for posttraumatic stress, depression, and risk for suicidal ideation among Air Force personnel. *Journal of Contextual Behavioral Science* 4(4), 263–268.

[ref15] Byrne BM (1994) Testing for the factorial validity, replication, and invariance of a measuring instrument: A paradigmatic application based on the Maslach Burnout Inventory. *Multivariate Behavioral Research* 29(3), 289–311. 26765139 10.1207/s15327906mbr2903_5

[ref16] Caricati L, D’Agostino G, Sollami A, et al. (2022) A study on COVID-19-related stigmatization, quality of professional life and professional identity in a sample of HCWs in Italy. *Acta Bio Medica: Atenei Parmensis* 93(2), 2. 10.23750/abm.v93iS2.12613 PMC9534206 35545987

[ref17] Carré A, Stefaniak N, D’ambrosio F, et al. (2013) The Basic Empathy Scale in Adults (BES-A): Factor structure of a revised form. *Psychological Assessment* 25(3), 679. 10.1037/a0032297 23815121

[ref18] Chan WCH, Fong A, Wong KLY, et al. (2016) Impact of death work on self: Existential and emotional challenges and coping of palliative care professionals. *Health and Social Work* 41(1), 33–41. 26946884 10.1093/hsw/hlv077

[ref19] Cohen S, Gianaros PJ and Manuck SB (2016) A stage model of stress and disease. *Perspectives on Psychological Science* 11(4), 456–463. 27474134 10.1177/1745691616646305 PMC5647867

[ref20] Conversano C, Ciacchini R, Orrù G, et al. (2020) Mindfulness, compassion, and self-compassion among health care professionals: What’s new? A systematic review. *Frontiers in Psychology* 11, 1683. 10.3389/fpsyg.2020.01683 PMC7412718 32849021

[ref21] Correia I and Almeida AE (2020) Organizational justice, professional identification, empathy, and meaningful work during COVID-19 pandemic: Are they burnout protectors in physicians and nurses? *Frontiers in Psychology* 11, 3545. 10.3389/fpsyg.2020.566139 PMC7759469 33362629

[ref22] Cross LA (2019) Compassion fatigue in palliative care nursing: A concept analysis. *Journal of Hospice & Palliative Nursing* 21(1), 21. 10.1097/NJH.0000000000000477 PMC6343956 30608916

[ref23] Crosswell AD and Lockwood KG (2020) Best practices for stress measurement: How to measure psychological stress in health research. *Health Psychology Open* 7(2), 2055102920933072. 10.1177/2055102920933072 PMC7359652 32704379

[ref24] de Boer AG, van Lanschot JJ, Stalmeier PF, et al. (2004) Is a single-item visual analogue scale as valid, reliable and responsive as multi-item scales in measuring quality of life? *Quality of Life Research* 13, 311–320. 15085903 10.1023/B:QURE.0000018499.64574.1f

[ref25] Diehl E, Rieger S, Letzel S, et al. (2020) Health and intention to leave the profession of nursing-which individual, social and organisational resources buffer the impact of quantitative demands? A cross-sectional study. *BMC Palliative Care* 19, 1–13. 32552671 10.1186/s12904-020-00589-y PMC7298824

[ref26] Duarte J and Pinto-Gouveia J (2017) The role of psychological factors in oncology nurses’ burnout and compassion fatigue symptoms. *European Journal of Oncology Nursing* 28, 114–121. 28478848 10.1016/j.ejon.2017.04.002

[ref27] Figley CR (1995). Compassion fatigue: Toward a new understanding of the costs of caring.

[ref28] Fillion L, Tremblay I, Truchon M, et al. (2007) Job satisfaction and emotional distress among nurses providing palliative care: Empirical evidence for an integrative occupational stress-model. *International Journal of Stress Management* 14(1), 1.

[ref29] Folkman S and Lazarus RS (1988) Coping as a mediator of emotion. *Journal of Personality and Social Psychology* 54(3), 466. 3361419

[ref30] Frache S, Balizet D, Simonin C, et al. (2020) Que font les médecins en unité de soins palliatifs? Étude descriptive de la pratique médicale dans une unité de soins palliatifs en France. *Médecine Palliative* 19(5), 282–287.

[ref31] Galiana L, Oliver A, Arena F, et al. (2020) Development and validation of the Short Professional Quality of Life Scale based on versions IV and V of the Professional Quality of Life Scale. *Health and Quality of Life Outcomes.* 18, 1–12. 33176807 10.1186/s12955-020-01618-3 PMC7656889

[ref32] Gamino LA and Ritter RH, Jr (2012) Death competence: An ethical imperative. *Death Studies* 36(1), 23–40. 24567993 10.1080/07481187.2011.553503

[ref33] Gentili C, Rickardsson J, Zetterqvist V, et al. (2019) Psychological flexibility as a resilience factor in individuals with chronic pain. *Frontiers in Psychology* 10, 2016. 10.3389/fpsyg.2019.02016 PMC6734029 31551871

[ref34] Grégoire S, Gagnon J, Lachance L, et al. (2020) Validation of the English and French versions of the multidimensional psychological flexibility inventory short form (MPFI-24). *Journal of Contextual Behavioral Science* 18, 99–110.

[ref35] Hayes SC, Luoma JB, Bond FW, et al. (2006) Acceptance and commitment therapy: Model, processes and outcomes. *Behaviour Research and Therapy* 44(1), 1–25. 16300724 10.1016/j.brat.2005.06.006

[ref36] Hayes SC, Strosahl KD and Wilson KG (1999) *Acceptance and Commitment Therapy*. Vol. 6 New York: Guilford press.

[ref37] Ho Chan WC and Tin AF (2012) Beyond knowledge and skills: Self-competence in working with death, dying, and bereavement. *Death Studies* 36(10), 899–913. 24563946 10.1080/07481187.2011.604465

[ref38] Jolliffe D and Farrington DP (2006) Development and validation of the Basic Empathy Scale. *Journal of Adolescence* 29(4), 589–611. 16198409 10.1016/j.adolescence.2005.08.010

[ref39] Kabat-Zinn J (2003). Mindfulness-based interventions in context: Past, present, and future.

[ref40] Kabat-Zinn J (2009) *Wherever You Go, There You Are: mindfulness Meditation in Everyday Life*. London: Hachette UK.

[ref41] Karasek R, Brisson C, Kawakami N, et al. (1998) The Job Content Questionnaire (JCQ): An instrument for internationally comparative assessments of psychosocial job characteristics. *Journal of Occupational Health Psychology* 3(4), 322. 10.1037//1076-8998.3.4.322 9805280

[ref42] Kashdan TB and Rottenberg J (2010) Psychological flexibility as a fundamental aspect of health. *Clinical Psychology Review.* 30(7), 865–878. 21151705 10.1016/j.cpr.2010.03.001 PMC2998793

[ref43] Kearney MK, Weininger RB, Vachon ML, et al. (2009) Self-care of physicians caring for patients at the end of life: “Being connected … a key to my survival. *Jama* 301(11), 1155–1164. 19293416 10.1001/jama.2009.352

[ref44] Keene EA, Hutton N, Hall B, et al. (2010) Bereavement debriefing sessions: An intervention to support health care professionals in managing their grief after the death of a patient. *Pediatric Nursing* 36(4), 185–190. 20860257

[ref45] McCloskey S and Taggart L (2010) How much compassion have I left? An exploration of occupational stress among children’s palliative care nurses. *International Journal of Palliative Nursing* 16(5), 233–240. 20679971 10.12968/ijpn.2010.16.5.48144

[ref46] McKenna M, Dempster M, Jarowslawska A, et al. (2022) Moderating the work distress experience among inpatient hospice staff: A qualitative study. *International Journal of Palliative Nursing* 28(6), 280–288. 35727835 10.12968/ijpn.2022.28.6.280

[ref47] Melvin CS (2015) Historical review in understanding burnout, professional compassion fatigue, and secondary traumatic stress disorder from a hospice and palliative nursing perspective. *Journal of Hospice & Palliative Nursing* 17(1), 66–72.

[ref48] Meyer EC, La Bash H, DeBeer BB, et al. (2019) Psychological inflexibility predicts PTSD symptom severity in war veterans after accounting for established PTSD risk factors and personality. *Psychological Trauma: Theory, Research, Practice, and Policy* 11(4), 383–390. doi:10.1037/tra0000358 30148370

[ref49] Neff KD (2003) The development and validation of a scale to measure self-compassion. *Self and Identity* 2(3), 223–250.

[ref50] O’brien RM (2007) A caution regarding rules of thumb for variance inflation factors. *Quality and Quantity* 41, 673–690.

[ref51] O’Mahony S, Gerhart JI, Grosse J, et al. (2016) Posttraumatic stress symptoms in palliative care professionals seeking mindfulness training: Prevalence and vulnerability. *Palliative Medicine* 30(2), 189–192. doi:10.1177/0269216315596459 26186929

[ref52] Orellana-Rios CL, Radbruch L, Kern M, et al. (2018) Mindfulness and compassion-oriented practices at work reduce distress and enhance self-care of palliative care teams: A mixed-method evaluation of an “on the job “program. *BMC Palliative Care* 17(1), 1–15. 10.1186/s12904-017-0219-7 PMC5501358 28683799

[ref53] Papworth A, Ziegler L, Beresford B, et al. (2023) Psychological well-being of hospice staff: Systematic review. *BMJ Supportive & Palliative Care* 13(e3), e597–e611. 10.1136/spcare-2022-004012 37098444

[ref54] Parola V, Coelho A, Cardoso D, et al. (2017) Prevalence of burnout in health professionals working in palliative care: A systematic review. *JBI Evidence Synthesis* 15(7), 1905–1933. 10.11124/JBISRIR-2016-003309 28708752

[ref55] Peters L, Cant R, Sellick K, et al. (2012) Is work stress in palliative care nurses a cause for concern? A literature review. *International Journal of Palliative Nursing* 18(11), 561–567. 23413505 10.12968/ijpn.2012.18.11.561

[ref56] Postmes T, Wichmann LJ, van Valkengoed AM, et al. (2019) Social identification and depression: A meta‐analysis. *European Journal of Social Psychology* 49(1), 110–126.

[ref57] Raes F, Pommier E, Neff KD, et al. (2011) Construction and factorial validation of a short form of the self‐compassion scale. *Clinical Psychology and Psychotherapy* 18(3), 250–255. 21584907 10.1002/cpp.702

[ref58] Samson T and Shvartzman P (2018) Association between level of exposure to death and dying and professional quality of life among palliative care workers. *Palliative and Supportive Care* 16(4), 442–451. 28641599 10.1017/S1478951517000487

[ref59] Sansó N, Galiana L, Oliver A, et al. (2015) Palliative care professionals’ inner life: Exploring the relationships among awareness, self-care, and compassion satisfaction and fatigue, burnout, and coping with death. *Journal of Pain and Symptom Management.* 50(2), 200–207. 25701688 10.1016/j.jpainsymman.2015.02.013

[ref60] Sarabia‐Cobo C, Pérez V, de Lorena P, et al. (2021) Experiences of geriatric nurses in nursing home settings across four countries in the face of the COVID‐19 pandemic. *Journal of Advanced Nursing* 77(2), 869–878. 33150622 10.1111/jan.14626

[ref61] Sinclair S (2011) Impact of death and dying on the personal lives and practices of palliative and hospice care professionals. *Canadian Medical Association Journal* 183(2), 180–187. 21135081 10.1503/cmaj.100511 PMC3033923

[ref62] Slocum-Gori S, Hemsworth D, Chan W, et al. (2013) Understanding compassion satisfaction, compassion fatigue and burnout: A survey of the hospice palliative care workforce. *Palliative Medicine* 27(2), 172–178. 22179596 10.1177/0269216311431311

[ref63] Stamm B (2010). The concise manual for the professional quality of life scale.

[ref64] Taherdoost H, Sahibuddin S and Jalaliyoon N (2022) Exploratory factor analysis; concepts and theory. *Advances in Applied and Pure Mathematics* 27, 375–382.

[ref65] Turner JC, Hogg MA, Oakes PJ, et al. (1987) *Rediscovering the Social Group: a Self-categorization Theory*. Oxford: Basil Blackwell.

[ref66] Veer IM, Riepenhausen A, Zerban M, et al. (2021) Psycho-social factors associated with mental resilience in the Corona lockdown. *Translational Psychiatry* 11(1), 67. 10.1038/s41398-020-01150-4 PMC7817958 33479211

[ref67] Williams VS, Morlock RJ and Feltner D (2010) Psychometric evaluation of a visual analog scale for the assessment of anxiety. *Health and Quality of Life Outcomes.* 8(1), 1–8. 20529361 10.1186/1477-7525-8-57 PMC2904728

[ref68] Wright DB, London K and Field AP (2011) Using bootstrap estimation and the plug-in principle for clinical psychology data. *Journal of Experimental Psychopathology* 2(2), 252–270.

[ref69] Zanatta F, Maffoni M and Giardini A (2020) Resilience in palliative healthcare professionals: A systematic review. *Supportive Care in Cancer* 28(3), 971–978. 31811483 10.1007/s00520-019-05194-1 PMC7223999

[ref70] Zeidan J (2012) Les différentes mesures du bien-être subjectif. *Revue Française D’économie* 123(3), 35–70.

[ref71] Zimmermann C, Swami N, Krzyzanowska M, et al. (2016) Perceptions of palliative care among patients with advanced cancer and their caregivers. *Canadian Medical Association Journal* 188(10), E217–E227. 27091801 10.1503/cmaj.151171 PMC4938707

