# Supplementary material for: Stress, mental health, and resources of palliative care professionals
Source: Palliat Support Care. 2025 Mar 28;23:e34. doi: 10.1017/S1478951524002050 (PMC13166306; doi:10.1017/S1478951524002050)
Supplement: Cuchet et al. supplementary material [file S1478951524002050sup001.docx]

Supplementary materials revised

Table S1. Internal consistency of the short Basic Empathy Scale (BES)

|  | Item-rest correlation | Cronbach’s α (if item is dropped) | McDonald’s ω (if item is dropped) |
| --- | --- | --- | --- |
| **emotional disconnection** |  |  |  |
| 1 My friends’ emotions don’t affect me much, R | .34 | .74 | .74 |
| 5 I don’t become sad when I see other people crying, R | .43 | .72 | .73 |
| 6 Other people’s feeling don’t bother me at all, R | .51 | .72 | .72 |
| 10 My friend’s unhappiness doesn’t make me feel anything, R | .45 | .72 | .72 |
| 11 I am not usually aware of my friends’ feelings, R | .33 | .74 | .74 |
| **Emotional contagion** |  |  |  |
| 2 After being with a friend who is sad about something, I  usually feel sad. | .50 | .72 | .72 |
| 4 I get caught up in other people’s feelings easily. | .56 | .71 | .71 |
| 8 I often become sad when watching sad things on TV or in  films. | .34 | .74 | .74 |
| 9 I often get swept up in my friends’ feelings. | .45 | .72 | .73 |
| **cognitive empathy** |  |  |  |
| 3 I can understand my friend’s happiness when she/he  does well at something. | .15 | .75 | .76 |
| 7 When someone is feeling ‘down’ I can usually understand  how they feel. | .28 | .74 | .75 |
| 12 I have trouble figuring out when my friends are happy, R | .20 | .75 | .75 |
| **Overall scale consistency** |  | **.75** | **.75** |
| Note. R: reverse scale items |  |  |  |

Table S2. Internal consistency of the FFMQ-15: 15-item Five-Facet Mindfulness Questionnaire

(attention to the present moment scale) at 15 and 14 items

|  | 15 items scale | | | 14 items scale (item 6 removed) | |
| --- | --- | --- | --- | --- | --- |
|  | Item-rest correlation | Cronbach’s α (if item is dropped) | McDonald’s ω (if item is dropped) | Cronbach’s α (if item is dropped) | McDonald’s ω (if item is dropped) |
| 1 When I take a shower or a bath, I stay alert to the sensations of water on my body. | .25 | .76 | .79 | .79 | .80 |
| 2 I’m good at finding words to describe my feelings | .46 | .74 | .77 | .77 | .79 |
| 3 I don’t pay attention to what I’m doing because I’m daydreaming, worrying, or otherwise distracted R | .35 | .75 | .78 | .78 | .79 |
| 4 I believe some of my thoughts are abnormal or bad and I shouldn’t think that way R | .41 | .75 | .77 | .77 | .79 |
| 5 When I have distressing thoughts or images, I “step back” and am aware of the thought or image without getting taken over by it. | .37 | .75 | .78 | .78 | .79 |
| 6 I notice how food and drink influence my thoughts, body sensations, and emotions | .02 | .79 | .80 | removed item | removed item |
| 7 I have trouble thinking of the right words to express how I feel about things R | .53 | .73 | .76 | .76 | .77 |
| 8 I do jobs or tasks automatically without being aware of what I’m doing R | .43 | .75 | .77 | .77 | .78 |
| 9 I think some of my emotions are bad or inappropriate and I shouldn’t feel them R | .53 | .74 | .76 | .76 | .77 |
| 10 When I have distressing thoughts or images, I am able just to notice them without reacting. | .27 | .76 | .79 | .79 | .80 |
| 11 I pay attention to sensations, such as the wind in my hair or sun on my face. | .38 | .75 | .78 | .78 | .80 |
| 12 Even when I’m feeling terribly upset, I can find a way to put it into words. | .56 | .73 | .76 | .76 | .78 |
| 13 I find myself doing things without paying attention R | .37 | .75 | .77 | .78 | .79 |
| 14 I tell myself I shouldn’t be feeling the way I’m feeling R | .45 | .74 | .77 | .77 | .78 |
| 15 When I have distressing thoughts or images, I just notice them and let them go. | .29 | .76 | .79 | .79 | .80 |
| **Overall scale consistency** |  | **.76** | **.79** | **.79** | **.80** |
| Note. R: reverse scale item | | | | | |

Table S3. Internal consistency of the Short Self Compassion Scale (SCS-SF) reduced to 7 items

|  | Item-rest correlation | Cronbach’s α (if item is dropped) | McDonald’s ω (if item is dropped) |
| --- | --- | --- | --- |
| 1 When I fail at something important to me, I become consumed by feelings of inadequacy (over identification) R | .69 | .82 | .82 |
| 2 When something painful happens I try to take a balanced view of the situation (mindfulness) | .32 | .87 | .87 |
| 3 When I’m feeling down, I tend to feel like most other people are probably happier than I am (isolation) R | .54 | .84 | .85 |
| 4 When I fail at something that’s important to me, I tend to feel alone in my failure (isolation) R | .67 | .82 | .83 |
| 5 When I’m feeling down, I tend to obsess and fixate on everything that’s wrong (over identification) R | .68 | .82 | .82 |
| 6 I’m disapproving and judgmental about my own flaws and inadequacies (self-judgment) R | .67 | .82 | .83 |
| 7 I’m intolerant and impatient towards those aspects of my personality I don’t like (self-judgment) R | .72 | .82 | .82 |
| **Overall scale consistency** |  | **.85** | **.85** |
| Note. R: reverse scale items |  |  |  |

Table S4. Internal consistency of the MPFI-24 scale (Grégoire) 12-item version and the reduced 11-item scale

|  | | MPFI-24 (Grégoire) 12-item version | | | 11-item version (item 7 removed) | | | |
| --- | --- | --- | --- | --- | --- | --- | --- | --- |
|  | | Item-rest correlation | Cronbach’s α (if item is dropped) | McDonald’s ω (if item is dropped) | Item-rest correlation | Cronbach’s α (if item is dropped) | McDonald’s ω (if item is dropped) | |
| **Flexibility subscale** | | |  |  |  |  |  | |
| 1 | Acceptance | .44 | .77 | .79 | .48 | .83 | .83 | |
| 2 | Contact with the present moment | .41 | .77 | .80 | .43 | .83 | .84 | |
| 3 | Defusion | .48 | .76 | .79 | .51 | .83 | .83 | |
| 4 | Self as context | .64 | .75 | .78 | .67 | .82 | .82 | |
| 5 | Contact with values | .58 | .75 | .78 | .61 | .82 | .82 | |
| 6 | Comitted action | .42 | .77 | .80 | .45 | .83 | .83 | |
| **Inflexibility subscale** | | |  |  |  |  | |  |
| 7 | Experiential avoidance R | -.33 | .84 | .84 | removed item | removed item | removed item | |
| 8 | Lack of Contact with the Present Moment R | .42 | .77 | .80 | .42 | .84 | .84 | |
| 9 | Self as content R | .54 | .76 | .79 | .53 | .83 | .83 | |
| 10 | Fusion R | .57 | .75 | .79 | .57 | .82 | .83 | |
| 11 | Lack of Contact with Values R | .49 | .76 | .80 | .49 | .83 | .83 | |
| 12 | Inaction R | .57 | .75 | .79 | .56 | .82 | .83 | |
| **Overall scale consistency** | |  | **.78** | **.81** |  | **.84** | **.84** | |
| Note. R: Reverse scale item | | | | | | | | |

Table S5. Internal consistency of the 5 VAS health measures

|  | Item-rest correlation | Cronbach’s α (if item is dropped) | McDonald’s ω (if item is dropped) |
| --- | --- | --- | --- |
| 1 Please indicate your level of life satisfaction over the past few weeks by moving the slider. | .65 | .79 | .79 |
| 2 Please indicate your level of anxiety over the past few weeks by moving the slider. R | .56 | .81 | .83 |
| 3 Please indicate your level of happiness over the past few weeks by moving the slider. | .66 | .78 | .79 |
| 4 Please indicate your level of depression over the past few weeks by moving the slider. R | .65 | .77 | .81 |
| 5 Please indicate your level of inner peace over the past few weeks by moving the slider. | .63 | .78 | .81 |
| **Overall scale consistency** |  |  |  |
| Note. R: reverse scale items |  | **.82** | **.84** |

Table S6. Internal consistency of the ProQOl scale

|  | Item-rest correlation | Cronbach’s α (if item is dropped) | McDonald’s ω (if item is dropped) |
| --- | --- | --- | --- |
| **compassion fatigue dimension** |  |  |  |
| 1 I think that I might have been affected by the traumatic stress of those I help | .47 | .81 | .81 |
| 4 I feel depressed as because of the traumatic experiences of the people I help | .67 | .78 | .79 |
| 8 As a result of my helping, I have intrusive, frightening thoughts | .52 | .80 | .81 |
| **Burnout dimension** |  |  |  |
| 2 I feel trapped by my job as a health professional | .64 | .78 | .79 |
| 6 I feel worn out because of my work as a helper | .64 | .78 | .80 |
| 7 I feel overwhelmed because the size of my workload seems endless | .49 | .81 | .81 |
| **Compassion satisfaction dimension** |  |  |  |
| 3 I like my work as a health professional R | .39 | .81 | .82 |
| 5 My work makes me feel satisfied R | .47 | .81 | .81 |
| 9 I am happy that I chose to do this work R | .41 | .81 | .82 |
| **Overall scale consistency** |  | **.82** | **.82** |
| Note. R: reverse scale items |  |  |  |
